# Strengthening Interactions between Statisticians and Collaborators: Objectives and Sample Sizes

**DOI:** 10.4172/2155-6180.1000e127

**Published:** 2014

**Authors:** Emily Van Meter, Richard Charnigo

**Affiliations:** 1 Department of Biostatistics, University of Kentucky, USA; 2 Department of Statistics, University of Kentucky, USA

## Background

Effective determinations of sample size require interaction between statisticians and their research collaborators who wish to initiate studies. The majority of grant applications, clinical trial protocols, and IRB submissions will not be approved unless there is some statistical justification for the planned sample size that is appropriate for the study’s primary objective [1]. However, obtaining a suitable sample size is non-trivial and may necessitate dynamic conversations regarding aims, objectives, study design, and future directions. Such conversations may address the types of data to be collected, analytic plans, and whether the specified endpoints and objectives will answer the goals of the study post-completion.

Beyond meeting the challenge of satisfying reviewers, a well-informed sample size justification will ensure reasonable precision in estimation and adequate statistical power for hypothesis tests. Such a justification is also made in the context of proposing a budget and assessing feasibility of the study design. The sample size must be sufficient to test the primary objective and large enough to obtain preliminary data for secondary objectives and correlative studies; but an excessive sample size may be seen as wasteful and perhaps even unethical, if unnecessarily many subjects are randomized to an ineffective treatment. This editorial, based in part on the authors’ own real-world experiences, is directed to statisticians and will highlight some important factors to consider and discuss with collaborators to ensure proper study design, endpoint collection, and sample size. Some such factors may be easy to overlook, even for a statistician, while some pertain to finding common ground with scientists whose statistical training may be limited.

## Outcomes and Objectives

All research proposals begin with aims. The primary objective will influence all aspects of a study, including but not limited to data collection, sample size, design, and analytic plans. Therefore, the primary objective is an excellent starting point for discussions between statisticians and collaborators, as specifying succinct aims and appropriate endpoints will drive the rest of the design process.

### Aims versus endpoints

Sometimes a collaborator may struggle with identifying aims and corresponding endpoints. An outcome measure, also called an endpoint, relates to the parameter of interest in a study aim; however, outcomes are not synonymous with aims. An outcome is a patient-level measure of effect. Ideally, endpoints should be valid and reliable, quantifiable, easy to observe, free of measurement error, capable of being observed independently of the treatment assignment, and clinically relevant [2]. Aims must be clear, concrete, and inclusive of outcomes that can be measured in a realistic timeframe. They must be more specific than whether the treatment “works”; one question to ask is, “How will you determine that treatment is effective and worth future research for your patient population?” Writing a hypothesis can also help to clarify an aim and guide the analytic plan. During an introductory meeting, a statistician may find it helpful to ask collaborators for a five-minute synopsis of their overall study goals. From that, a statistician may readily identify the overarching aims of the study and help the collaborator to word them precisely but succinctly. However, further information may be required to ascertain what type of data should be collected. Asking questions may help to clarify this. Examples include:

1. “How often and for how long will you follow up with your subjects to obtain endpoints?”
2. “How do you imagine the data looking at the end of the trial?”
3. “If your aim is to show that a particular treatment is better, what information do we need to collect to definitively achieve this aim?”

For oncological and other clinical trials, endpoints differ by each phase in drug development. Phase I trials aim to assess safety and identify an appropriate dose; consequently, the corresponding endpoint must be, or at least encompass, a toxicity outcome [3]. These studies are typically single arm, non-randomized trials whose results are analyzed mainly with descriptive statistics rather than formal hypothesis tests. Phase II studies look for hints of efficacy that would warrant Phase III trials. Phase II trials often assess surrogate endpoints, clinical response rates, and percentages of patients that “succeed” with the treatment, as defined for a particular trial prior to its initiation. They can be single arm studies with historical control comparisons, but recently there has been a call for randomized Phase II trials [4]. In Phase III trials, the goal is to show definitive clinical benefit with a head-to-head comparison involving at least two groups, which ordinarily requires time-to-event endpoints such as disease progression, mortality, heart attack or stroke, and so forth.

Statisticians can help investigators to ensure that their aims match the appropriate phase of drug development. In fact, statisticians may occasionally help collaborators planning a Phase II study to realize that a Phase I trial ought to be conducted first. This may be the case when, for example, the referenced dose is unsuitable for the targeted patient population or not yet tested in combination with another drug.

### Primary outcomes

Virtually all studies will have multiple questions; however, the primary question should be the most clinically important and will drive the study design. Ideally, a single response variable should be identified to answer the primary question [5]. If more than one is used, the probability of getting a nominally significant result by chance alone is increased, unless one incorporates a multiplicity correction; but then statistical power is sacrificed.

### Secondary aims and correlatives

Secondary aims are important but do not drive design. In phase I trials, these can include descriptions of pharmacokinetics, pharmacodynamics, or preliminary clinical responses. In phase II studies, secondary objectives can be to estimate overall survival, describe safety, evaluate changes in biomarkers, assess quality of life measures, or address subsidiary questions relating to a patient subgroup. However, all secondary aims should be specified before data collection and limited in number. Moreover, principles similar to those previously articulated still apply for writing secondary aims and determining corresponding outcomes. Relevant questions for a statistician to discuss with a collaborator may include:

1. “What is the biologic rationale for these additional aims?”
2. “Will samples collected come from biopsy tissue or blood, and if so is collection of patient samples in a pre-post design feasible?”
3. “Will the additional effort to acquire these endpoints add to the scientific knowledge gained from your study, and is the effort worth the time, money, and resources?”

Additionally, sketching an excel spreadsheet shell may be helpful to see what data the investigators plan to collect (regardless of what data management program will be used). Since researchers are generally familiar with excel, such a sketch can provide a springboard for discussing which outcomes are most critical, how often they will be measured, how they will be categorized, and so forth. This step can help researchers to visualize how their data will look at the end of the trial and may identify data collection needs that have been overlooked.

## Sample Size Considerations

### The basics

After the aims are specified and corresponding endpoints identified, sample size calculations may commence; however, there are many factors to consider. The three basic components include alpha, beta, and delta (clinically relevant difference), as detailed in Table 1. Alpha is chosen a priori and typically set to 0.05. In phase II clinical trial or pilot studies, some investigators will set alpha=0.10 or 0.15, although this is not as common [4].

Power is equal to 1-beta and is typically set to 80% (or sometimes 90% for confirmatory studies). If power is too low, then there is little chance of rejecting the null even if the alternative is true, which is potentially disastrous given that substantial resources are often invested in a study. Assuming that the clinically relevant difference has not been mis-specified (see next paragraph), the cause of low power is inadequate sample size [6]. Perhaps counter intuitively, one may also have power that is too high. For instance, doubling the sample size starting from 50% power may lead to 79% power, whereas doubling the sample size starting from 98% power may yield 99.99% power. Since one was already very unlikely to commit a Type II error at 98% power, the doubling of that sample size may be seen as wasteful and even unethical.

**Figure 1:**
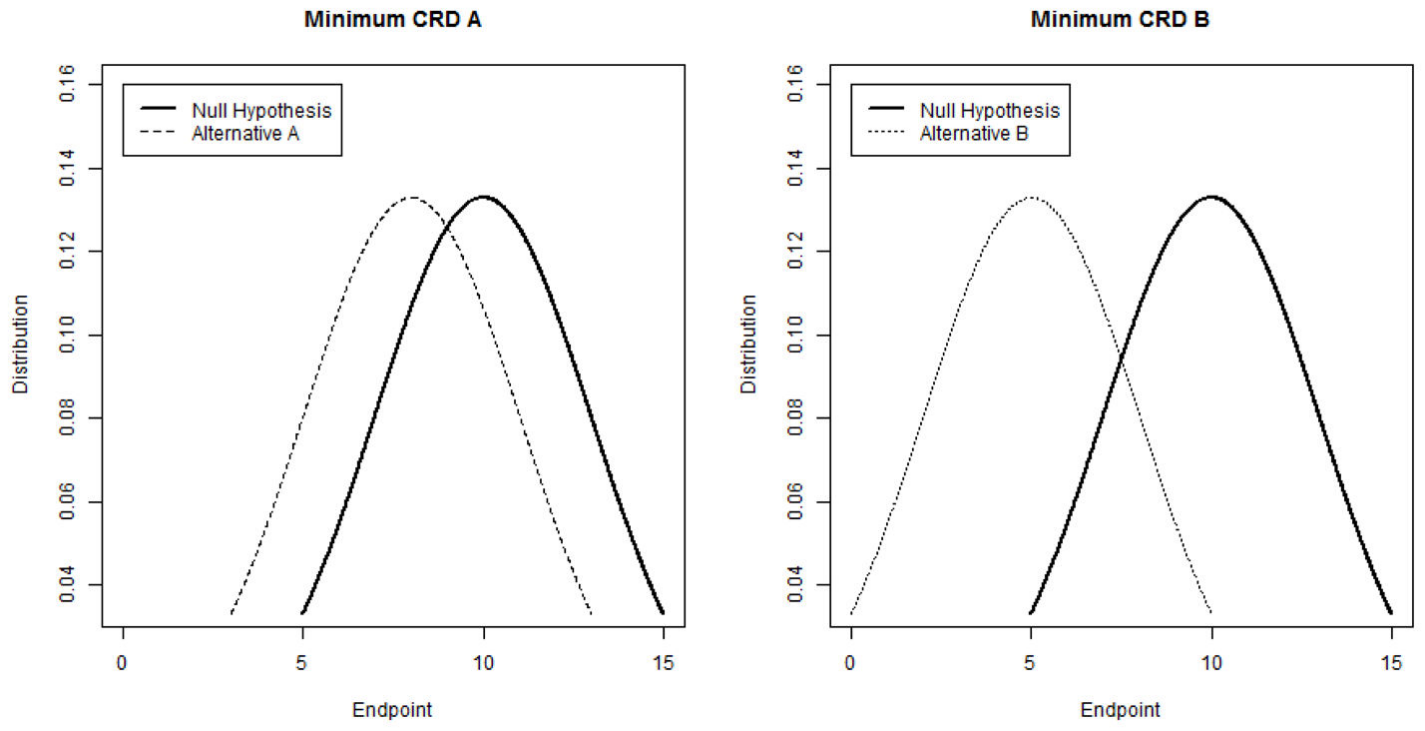
Example of bell curves to visualize minimum CRD.

The minimum clinically relevant difference (CRD or delta) is often the most difficult piece of information to obtain pre-study. The minimum CRD relates specifically to the primary endpoint. Depending on the variable type, this can be a percentage, mean (expressed relative to a corresponding standard deviation), median (expressed relative to a corresponding range or inter-quartile range), or hazard ratio. The minimum CRD can sometimes be informed by pilot data or other published studies, but in any case there must be a clinical justification: what treatment effect would ultimately change clinical practice [7]? Importantly, statistical significance does not imply clinical significance, and therefore the minimum CRD may not be what was observed previously. Of course, investigators sometimes have little or no pilot data to inform specification of the minimum CRD. In such cases, a statistician may aid them by sketching suitable diagrams. For instance, Figure 1 depicts two partially overlapping bell curves corresponding to two different choices of the minimum CRD for a normally distributed endpoint. Then investigators may select which, if either, diagram they think may represent a realistic expectation for the data they intend to collect.

### Types of endpoints

Among several other possibilities, endpoints can be continuous, binary, or time-to-event. There are pros and cons to each type, and some study designs will naturally favor one type over the others. Nonetheless, this choice will influence both the sample size calculation and the selection of statistical methodology for the data analysis plan. Categorical data are easy to describe and interpret, but caution is required because such data usually require greater sample sizes. For example, a binary outcome can only be a “0” or a “1”, so variability between groups is thereby limited. In fact, there is usually a substantial loss of power when inherently continuous data are categorized. Additionally, there is potential ambiguity in the selection of cut points for such categorizations, which may preclude comparisons across or meta-analysis of related studies [8].

### Number of groups

When there are two or more groups, the investigators need to decide upon randomization, blinding, and treatment allocation. Regarding the latter, the most practical option is usually 1:1 group allocation, as this generally yields optimal or near-optimal power for the total sample size. However, there are some situations where assigning more subjects to a new procedure may enhance enrollment and mitigate ethical concerns about exposing subjects to a riskier or potentially less effective treatment.

For continuous outcomes with at least three groups, there are sample size formulas for the ANOVA F test. However, the investigator’s true interest usually lies in the post-hoc comparisons between specific groups. A statistician calculating a sample size to power a post-hoc comparison must take into account the Bonferroni or other adjustment for multiple such comparisons. This is unfortunately easy to overlook, and failing to take into account such an adjustment may result in grossly underpowered comparisons between specific groups.

### Withdrawals and non-compliance

Missing outcome data may arise when subjects are lost to follow up, withdraw consent, die, or refuse to answer. There are also situations in which subjects (or even study personnel) do not comply with the treatment or protocol. If missing outcome data are anticipated, then sample size estimates should be adjusted accordingly. For instance, if 20% of subjects are anticipated to drop out, then sample size estimates should be inflated by a minimum of 25%. In such a case, a statistician may also wish to incorporate some type of imputation into the data analysis plan.

### Parametric assumptions

Also important, both to sample size calculations and to development of the data analysis plan, are discussions of assumptions for parametric tests. This is because nonparametric tests usually have less statistical power than parametric tests. Thus, choosing a sample size in anticipation of a parametric test and discovering later that a nonparametric test is required will lead to an underpowered comparison. Reviewing literature from other studies that use similar outcomes can be very helpful to assess whether the need for a nonparametric test is at least somewhat likely. If so, then inflating the sample size by 10–15% a priori may be a good idea.

Even for parametric tests, there may be some pitfalls to using standard sample size formulas. For instance, when employing a T test to compare two groups on a continuous outcome, the usual sample size formula actually involves quantiles of a Z distribution. They are meant to approximate quantiles of a T distribution, but the degrees of freedom for that T distribution are unknown since they depend on the sample size that one is trying to determine. If investigators hope to justify a sample size in the neighborhood of 5 to 15, this Z approximation to a T quantile may result in unexpectedly low power. As a practical rule of thumb, adding two subjects per group to whatever answer is obtained with the Z approximation works surprisingly well.

## Conclusion

**Figure 2:**
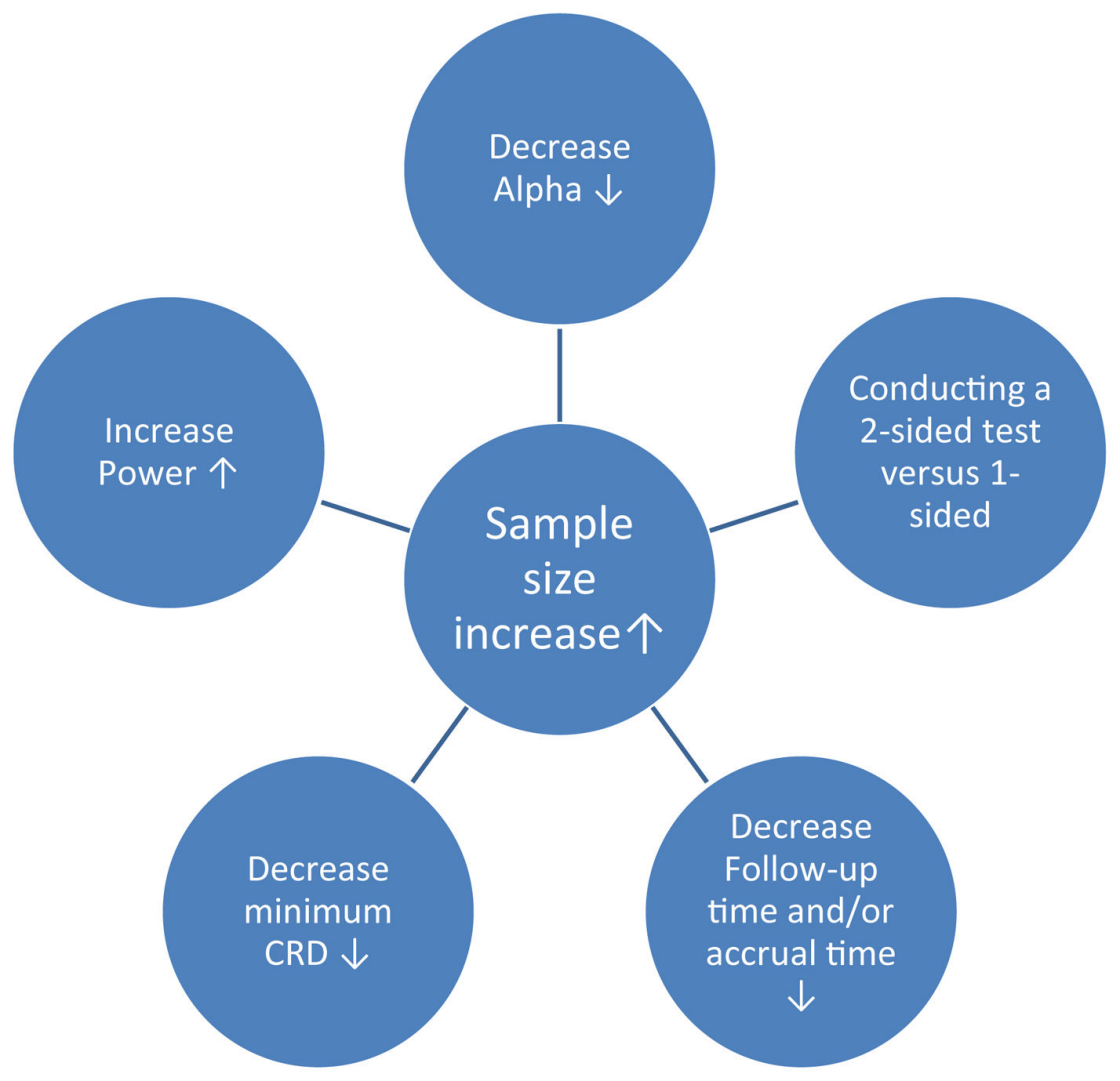
Factors that cause sample size to increase when keeping all other

The study aims and corresponding outcome measurements influence all other aspects of study design, including sample size calculations and formulation of a statistical analysis plan. As shown in Figure 2, altering simply one factor within a study design can cause the sample size to change significantly. Writing aims well is challenging because an investigator must convince readers that the aims are both achievable and worthwhile. A further challenge emerges because the process of study design is not always linear. For instance, upon completion of a sample size calculation, an investigator may change his/her aims to avoid prohibitive expenses [2]. Likewise, accrual limitations may have implications for study design. Ongoing interactions among the entire research team, including the statistician(s), can provide solutions, or at least alternatives, when difficulties arise. Beyond that, statisticians must also help investigators to avoid common errors, such as those described above and one other noted by Kramer and Kupfer [9]: “One common mistake in [study design] is guaranteeing adequate power, not at or above the threshold of clinical significance, but at or above the desired or hoped-for effect size or one based on very optimistic, underpowered, pilot studies.” Taking time prior to study initiation to discuss objectives and sample sizes is critical, since a poorly designed study cannot be fixed and may not even be salvageable post-completion [10].

## Figures and Tables

**Table 1:** Basic components for sample size calculations.

	Definition	What does this mean clinically?
Alpha-α (Type I Error probability) Significance Level of a test	Probability of rejecting the null hypothesis when it is true	Superiority Setting-Falsely claiming a difference Non-inferiority Setting-Falsely claiming a similarity
Beta-β (Type II Error probability) Complementary to power of a test	Probability of failing to reject a false null hypothesis	Superiority Setting-Falsely claiming no difference Non-inferiority Setting-Falsely claiming a difference
Delta-δ (Minimum Clinically Relevant Difference or CRD)	What is the minimum treatment effect that would ultimately change clinical practice?	Superiority-lower bound on efficacy advantage of treatment Non-inferiority-upper bound on efficacy disadvantage of treatment
